# Hardness, Modulus, and Refractive Index of Plasma-Assisted Atomic-Layer-Deposited Hafnium Oxide Thin Films Doped with Aluminum Oxide

**DOI:** 10.3390/nano13101607

**Published:** 2023-05-10

**Authors:** Mikk Kull, Helle-Mai Piirsoo, Aivar Tarre, Hugo Mändar, Aile Tamm, Taivo Jõgiaas

**Affiliations:** Department of Materials Science, Institute of Physics, University of Tartu, W. Ostwald Str. 1, EE50411 Tartu, Estoniahugo.mandar@ut.ee (H.M.);

**Keywords:** atomic layer deposition, thin film, mechanical properties, nanoindentation, hardness, modulus, refractive index

## Abstract

Coatings with tunable refractive index and high mechanical resilience are useful in optical systems. In this work, thin films of HfO_2_ doped with Al_2_O_3_ were deposited on silicon at 300 °C by using plasma-enhanced atomic layer deposition (PE-ALD). The mainly amorphous 60–80 nm thick films consisted Al in the range of 2 to 26 at.%. The refractive indexes varied from 1.69 to 2.08 at the wavelength of 632 nm, and they consistently depended on the composition. The differences were higher in the UV spectral region. At the same time, the hardness of the films was from 12–15 GPa; the modulus was in the range of 160–180 GPa; and the mechanical properties did not have a good correlation with the deposited compositions. The deposition conditions, element contents, and refractive indexes at respective wavelengths were correlated. The results indicated that it is possible to tune optical properties and retain mechanical properties of atomic layer-deposited thin films of HfO_2_ with Al_2_O_3_ as doping oxide. Such films could be used as mechanically resilient and optically tunable coatings in, for instance, micro- or nano-electromechanical systems or transparent displays.

## 1. Introduction

Hafnium oxide is a scientifically and technologically important material. It has been researched or applied as an optical coating, a dielectric material in capacitors, transistors, and processors; a material in bio-sensors, a resistive switching medium, ferroelectric or pyroelectric material, etc. [1,2,3,4,5,6,7,8,9,10,11,12,13,14,15]. Among other applications, such coatings could be used as optical interference filter material in lab-on-chip for targeted biological sensors [16]. HfO_2_ has been proposed as an environmentally protective and reflectivity-enhancing coating for aluminum micromirrors [17]. A thin film applied to an optical system is expected to possess certain mechanical stability, or, generally, resilience [18].

Several different methods have been used to produce HfO_2_. For instance, hydrothermal or wet-chemical routes [8,9,19], magnetron sputtering [3,4,10,15,20] and atomic layer deposition (ALD) [1,5,7,8,21,22]. It has appeared as fibers [9], nanoparticles [19], and thin films [1,3,4,5,8,10,12]. HfO_2_ can possess amorphous [4,23] or nanocrystalline forms [3,8,10,19,21]. HfO_2_ has also been produced in various doped variants with Si, Zr, Y, or Al as the dopant [8,12,23,24,25,26,27].

Different precursor combinations have been used for ALD of hafnia. Typical are halides of Hf [21,22,27,28,29] and metal-organic precursors, such as amino compounds [1,23,26,30]. Water vapor, ozone, O_2_, or O_2_ plasma have been used as oxygen sources [7,22,23,26,27,30]. The variety in crystallographic phases of ALD hafnia includes (X-ray) amorphous, monoclinic, tetragonal, and cubic phases [8,21,27,29,30,31]. The phase composition tends to depend on the used precursors and deposition temperature. Other phases, such as orthorhombic, have been reported for Si-doped and post-deposition annealed HfO_2_ thin films [23].

HfO_2_ has good transparency in the infrared to ultraviolet spectral range [32]. The transmittance of ALD hafnia has been shown to be near 80%, but it depends on the film growth temperature [1,29]. The refractive index for ALD hafnium oxide is usually above 2. The thin-film growth temperature has a minute influence on the refractive index [21].

Hardness values of HfO_2_ on silicon have been reported to be 8.3–9.7 GPa and 9.5 ± 2 GPa [30,33]. It has also been shown that rapid annealing can increase the hardness up to 14.4 GPa [30]. As for the elastic modulus, the results varied from 163–165 GPa to 220 ± 40 GPa [30,33]. In these examples, the film thicknesses ranged from 60 to 100 nm. HfO_2_ was deposited on silicon substrates using tetrakisdimethylamidohafnium/water and tetrakisethylmethylaminohafnium/ozone processes at 300 °C or less. In a previous work, HfO_2_ on glass substrates (deposited using a HfCl_4_/water process) had a modulus of 111(11) GPa and a hardness of 9.1(0.7) GPa. The thin-film thickness was approximately 160 nm [34].

Despite the fact that for years ALD has been used to deposit HfO_2_ and its doped versions, including Al as the dopant, there are fewer reports on mechanical properties of such films. In a previous work about Al_2_O_3_-doped ZrO_2_, it was shown that Al_2_O_3_ can induce and stabilize a metastable tetragonal phase of zirconia [35]. The same has been claimed about Al_2_O_3_-doped HfO_2_ [8]. Metastable phases might have higher hardness and modulus and, therefore, could improve the mechanical resilience of thin films.

In the current work, plasma-enhanced atomic layer deposition was used to produce HfO_2_ thin films doped with Al_2_O_3_. The aim was to determine the mechanical and optical properties of the films in relation to their elemental and phase compositions. Such thin films could be used as functional coatings in micro- or nano-electromechanical devices [36,37].

## 2. Materials and Methods

The HfO_2_ and Al_2_O_3_ thin films were deposited using an R200 Advanced (Picosun, Masala, Finland) ALD reactor equipped with a load-lock and oxygen plasma generator. Hf was deposited from tetrakisethylmethylaminohafnium (TEMAH, 99% purity, Strem, Bischheim, France), and aluminum was deposited from trimethylaluminum (TMA, 99% purity, Volatec, Porvoo, Finland). The pulse sequence for HfO_2_ deposition was 0.3/4/15/4 s (TEMAH/N_2_/O_2_ plasma/N_2_), and for Al_2_O_3_ it was 0.1/4/15/4 s (TMA/N_2_/O_2_ plasma/N_2_). The substrate Si (100) temperatures during depositions were 300 °C; two samples were deposited at 200 °C and 400 °C. The total cycle count was 700 cycles, or the closest possible to that. The deposition rate of HfO_2_ was 0.113 ± 0.002 nm/cycle, and that for Al_2_O_3_ was 0.091 ± 0.002 nm/cycle.

In the following text, HfO_2_ is abbreviated as H and Al_2_O_3_ is abbreviated as A. The respective number in front of the designation is the deposition cycle count of a single oxide. The first number in front of the parenthesis indicates the repetition of hafnia and alumina cycles (sometimes called super-cycles). Also, samples designated as 233 × (1H + 2A) and 233 × (2H + 1A) were deposited and measured after the first batch of samples had been characterized, aiming to verify the developed model for refractive index calculation with regard to elemental compositions.

The crystallographic phase composition and the thickness of the films were determined using X-ray diffraction (XRD) or reflectivity (XRR) methods with a diffractometer (Rigaku SmartLab™, Rigaku, Tokyo, Japan) working at 8.1 kW (Cu Kα radiation, λ = 0.154178 nm). An asymmetric 2θ-scan at a fixed grazing incidence (GIXRD) angle of *ω* = 0.42° was used for phase composition analysis. GlobalFit 2.0 was used for XRR model fitting. The element content was measured using X-ray fluorescence (Rigaku ZSX-400).

A spectroscopic ellipsometer (Semilab Sopra GES-5E, Semilab, Budapest, Hungary) with arms at 70 degrees, was used to measure thin film refractive indexes and thicknesses. Since the measured material systems are simple enough (extinction coefficients were *k* = 0), a Sellmeier double-parameter model was used for theoretical fitting (WinElli II v2.2.0.7 software, Semilab, Budapest, Hungary) of the measurements [38]. The worst fit had a coefficient of determination of R^2^ = 0.989.

The hardness and elastic modulus of the Al_2_O_3_-doped HfO_2_ films were investigated by using nanoindentation (Bruker Hysitron Triboindenter TI 980, Billerica, MA, USA; Minneapolis, MN, USA). The samples were measured using a Berkovich diamond tip. The device was calibrated using a fused silica standard (Bruker) with a defined hardness of 9.25 GPa and a reduced modulus of 69.6 GPa. The 20 calibration measurements in dynamic mode (continuous stiffness measurements) and subsequent testing on the silica indicated that the valid displacement range was approximately 8 nm to 75 nm. A single measurement in dynamic mode includes 60 separate data points. The standard deviations for a single measurement remained near 3 GPa for modulus and 0.3 GPa for hardness at displacements of approximately 10 nm. The deviations were somewhat reduced towards higher displacements. Thirty separate continuous stiffness measurements were performed on each prepared sample. Any low-quality measurements were removed, reducing the count of acceptable measurements by 3 to 8 measurements. Scanning probe microscopy (SPM) was performed with the same device using the same Berkovich-type diamond tip for probing. In addition to SPM images, a scanning electron microscope (SEM, FEI Helios™ NanoLab 600 DualBeam, FEI, Hillsboro, OR, USA) was used to investigate the surface morphology.

## 3. Results

### 3.1. The Element Content

The X-ray fluorescence analysis (Table 1) showed that there was no residual nitrogen detected at trustable levels in the thin films. The carbon content was, in most cases, near the respective detection limit, but only the films deposited at 200 °C and 400 °C had a carbon level 4–5 times higher than the limit; for the latter, the detection limit was 0.2 and the measure was 0.83 µg/cm^2^. Therefore, the carbon content of those films could be considered trustworthy. The noticeable carbon levels in 200 °C and 400 °C deposited films could come, respectively, from incomplete reactions at 200 °C or some thermal decomposition of TEMAH or TMA at 400 °C. However, as the thin films deposited at these temperatures actually did not create any remarkable variety in any of the discussed properties, most of the attention was later turned to samples deposited at 300 °C.

The amount of Al atoms in the first deposited batch varied from 2 at.% up to 26 at.% out of total amount of elements (Table 1). The error for an element’s content is given as plus–minus the triple of the respective detection limit. Checking the stoichiometry indicated that, in pure Al_2_O_3_, the ratio of O/Al was 1.5, which can be considered a fully stoichiometric oxide. Pure HfO_2_ had some excess of oxygen with an O/Hf ratio of 2.3, which is approximately 15% more oxygen than stoichiometrically expected. The samples with alumina addition had O/Hf ratios, mostly in the 2.1–2.2 range. The actual reason for such an inconsistency was not investigated but taken as is. The excess oxygen presence in the same precursors has been reported in a previous study [31], where the O/Hf ratio varied from 2.0 to 2.2 and depended on the deposition temperature. The ratio of 2.0 was obtained for samples deposited at 350 °C. At the same time, it was shown that the deposition rate increased at higher substrate temperatures due to some pyrolysis of the Hf precursor. In another study, it was shown that HfO_2_, using the same ALD process but deposited at 160 °C, had over 10 at.% of impurities [39]. That amount did not change during post-deposition annealing (in nitrogen or air) at 300 °C. X-ray photospectroscopy showed that the films had a certain amount of oxygen bound to hydrogen and carbon. During annealing in air, the O/Hf ratio had an increasing tendency, over the stochiometric value (approx. 2.05). Sub-stochiometric HfO_2_ from tetrakis(dimethylamino)hafnium and O_2_ plasma, deposited at 250 °C, has also been reported [40]. Therefore, with this precursor combination, some excess elements and imbalanced ratios of those can be expected.

The anion/cation ratio in metal oxides is important. Copper oxides are a typical example. CuO is an n-type conductor, whereas Cu_2_O is a p-type conductor [41]. As the O/Cu ratios change from 1 (CuO) to 0.75 (Cu_4_O_3_) to 0.5 (Cu_2_O), the optical band gaps change by 2.11 eV, 1.34 eV, or 2.47 eV (depending on the analysis method) and 2.45 eV, respectively. A similar trend occurs in electrical resistivity, which reduces in the same row as 10 × 10^8^ µΩcm, 6.2 × 10^8^ µΩcm, and 3 × 10^8^ µΩcm, respectively [42]. In the case of HfO_2_ thin films, Hildebrandt et al. have shown that the band gap for a stochiometric compound is 5.7 eV, but it reduces if oxygen vacancies or interstitials are present [43]. They achieved p-type conductivity in oxygen-vacant HfO_2_ thin films with a band gap down to 4.5 eV and resistivity of 400 µΩcm. Unfortunately, they did not report the elemental composition of their thin films or point out any other influence on properties caused by oxygen interstitials.

### 3.2. Film Thickness and Phase Composition

#### 3.2.1. Film Thickness, Density, and Roughness

**Figure 1:**
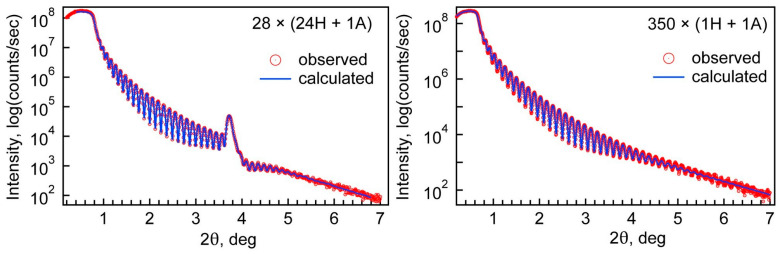
Examples of XRR measurements and fitting results.

The results of film thickness and density are summarized in Table 2. Examples of XRR measurement and fitting results can be seen in Figure 1. A set of XRR measurements and respective fits can be found in Supplementary part S1 (Figure S1). The density value for pure hafnia corresponds to the density of monoclinic HfO_2_, the phase confirmed by the diffraction pattern. The alumina density corresponds approximately to a previously published value [35]. The deviations for thickness values were 1 nm or less and 0.2 g/cm^3^ or less for density. Roughness values for alumina and doped films were approximately 1 nm and approximately 3.5 nm for pure HfO_2_.

Table 2 indicates that the deposition rate of HfO_2_ was 0.113 ± 0.002 nm/cycle and that for Al_2_O_3_ was 0.091 ± 0.002 nm/cycle.

#### 3.2.2. Phase Composition

**Figure 2:**
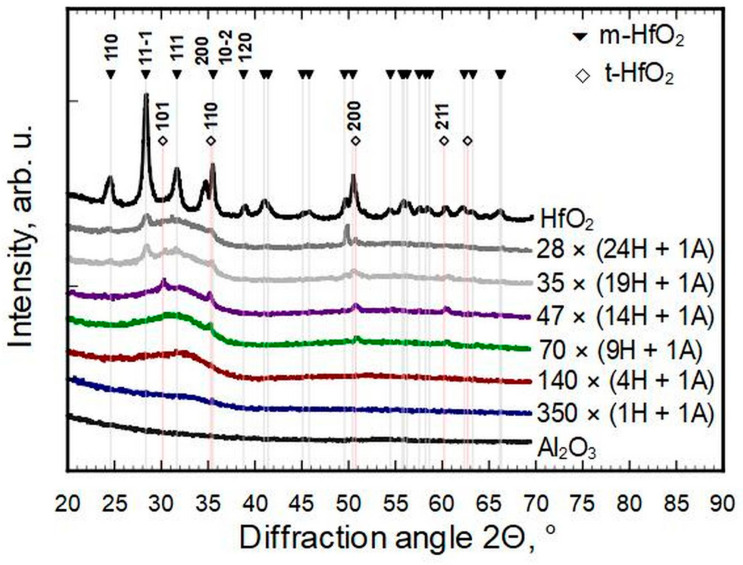
Diffraction patterns of thin films deposited at 300 °C.

The HfO_2_ diffraction pattern shows that it deposited mainly in the monoclinic phase (ICDD PDF-2 card #01-075-6426, Figure 2).

**Figure 3:**
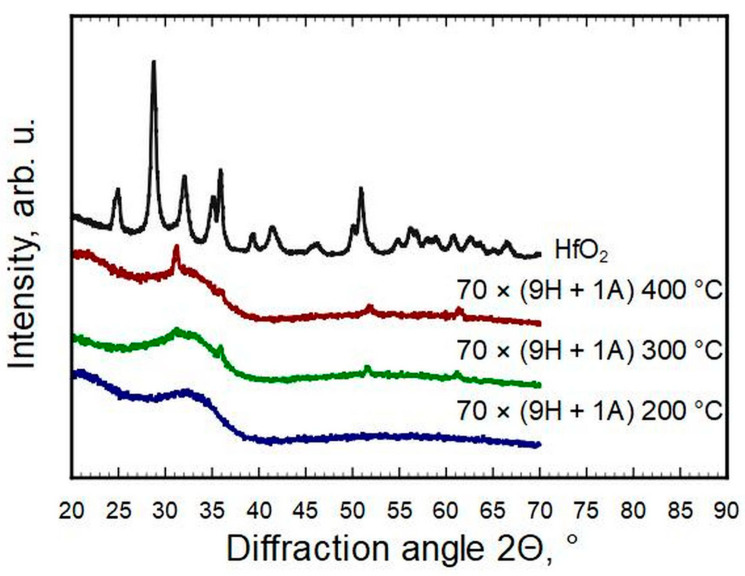
Phase composition evolution for the same deposition scheme but different growth temperatures.

Only a few reflections could be observed for thin films with a hafnia to alumina cycle ratio of 24 ÷ 1 to 9 ÷ 1. All four reflections on the patterns of samples 47 × (14H + 1A) and 70 × (9H + 1A) match with the strongest reflections of cubic or tetragonal metastable phases of hafnia (ICDD PDF-2 cards #53-0560 and #01-078-5756, correspondingly). The same could be claimed for 70 × (9H + 1A) deposited at T_g_ = 400 °C (Figure 3). The strongest reflections of monoclinic hafnia were present in the case of samples 35 × (19H + 1A) and 28 × (24H + 1A). The metastable phases of samples 47 × (14H + 1A) and 70 × (9H + 1A), detectable by a reflection at 2Θ ≈ 30.2° and indexed as 111 of the cubic or 101 of the tetragonal HfO_2_ phase, disappeared when the concentration of hafnium increased or decreased. The samples with an Al content of ≈6 at.% and more were X-ray amorphous and did not show any diffraction reflections. It was undetermined how much of the amorphous nature was caused by interstitial oxygen. The pure HfO_2_ pattern indicates a reasonably well-crystallized thin film, even though it had the highest O/Hf ratio, but adding Al in a few percent levels changed that remarkably.

### 3.3. Refractive Index

**Figure 4:**
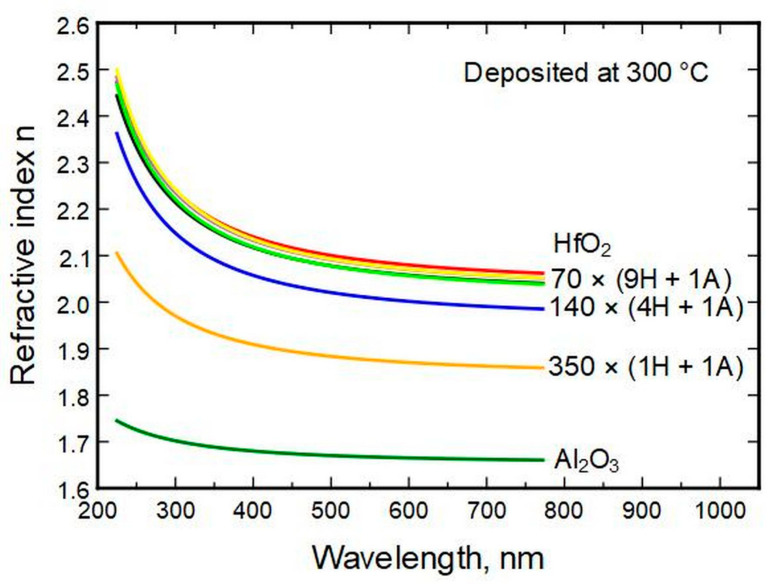
Refractive index calculated from ellipsometry data in relation to the cycles of HfO_2_ and Al_2_O_3_.

Spectroscopic ellipsometry (SE) allows the determination of the refractive indexes *n* of the thin films for a range of wavelengths. The measurements show that most of the change in *n* between wavelengths of 225 and 770 nm (photon energies of 5.5–1.6 eV) appears for thin films with HfO_2_ to Al_2_O_3_ cycle ratios of 1 ÷ 1 and 4 ÷ 1 (Figure 4). The difference is marginal between pure HfO_2_ and films with HfO_2_ cycle ratios of 9 and up. Throughout the compositions, the refractive indexes change consistently, with alumina possessing the lowest and hafnia the highest values. (See also Supplementary part S2, Figure S2)

Sun et al. have shown that the refractive index does not depend much on the O/Hf ratio [44]. Their samples had O/Hf ratios ranging from 1.98 to 2.09, and the highest difference in refractive indexes was approximately 0.02 at 775 nm. They also showed that the extinction coefficient *k* is 0 for the samples, which is a presumption in the discussion below.

**Figure 5:**
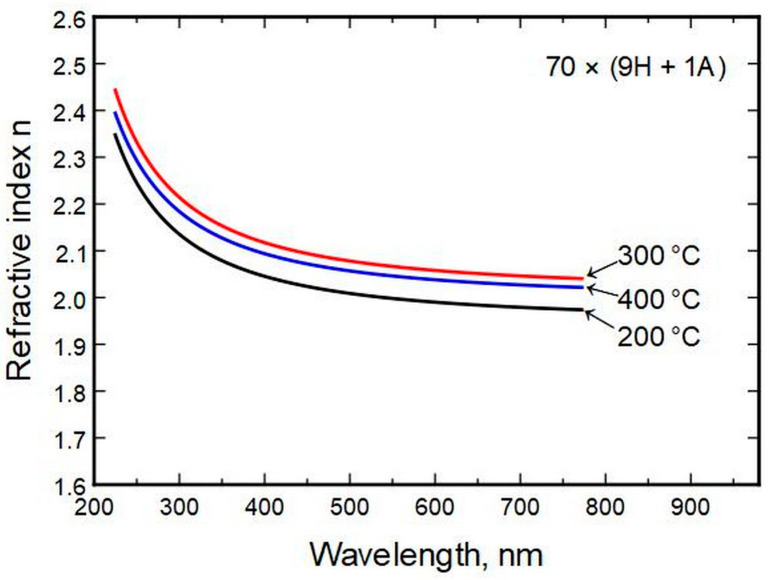
Refractive index relation to thin film deposition temperature.

Deposition temperature had a low effect on the refractive index compared to composition change (Figure 5).

### 3.4. Hardness and Modulus of Thin Films

#### 3.4.1. The Substrate and Single Oxides

The nanoindentation of Si (100) indicated that the substrate hardness was approximately 13 ± 1 GPa and the (reduced) modulus was 145 ± 10 GPa.

**Figure 6:**
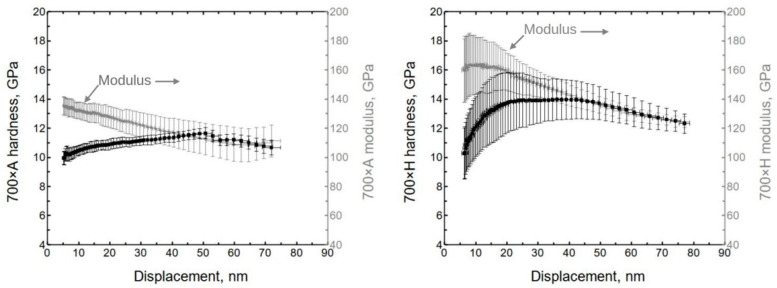
Hardness and modulus of Al_2_O_3_ and HfO_2_ on silicon deposited at 300 °C, as measured by nanoindentation.

The alumina indentation results show the non-uniform behavior of the material system (Figure 6). Initially, the hardness was approximately 10.5 ± 0.8 GPa, and the (reduced) modulus was 137 ± 7 GPa. (Hereafter, the values are given at ≈10 nm of displacement, if not stated otherwise.) These values are similar to previously published results [35].

The initial hardness of hafnia was approximately 10.5 ± 1.5 GPa, and the (reduced) modulus was 160 ± 20 GPa (Figure 6). The initial lower hardness value might be influenced by surface roughness since the X-ray reflectivity showed hafnia to have the highest roughness.

#### 3.4.2. The Doped Films

**Figure 7:**
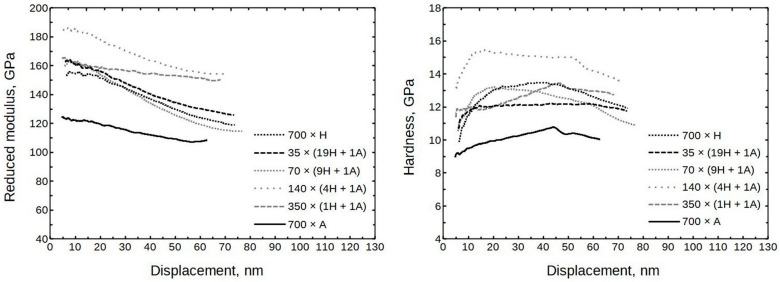
Averaged modulus and hardness values of Al_2_O_3_ doped HfO_2_ thin films as measured by nanoindentation.

The averaged moduli and hardness values are presented in Figure 7. (The full set is presented in Supplementary part S3, Figure S3) It can be seen that the moduli were in the approximate range of 150 to 190 GPa and the hardness values ranged from 11 to 14 GPa. Somewhat exceptional is sample 140 × (4H + 1A) with a hardness value of approximately 15 GPa.

The growth temperature did not have a large effect on the modulus for samples deposited at 300 °C and 400 °C (formula 70 × (9H + 1A)). At 10 nm of displacement, the moduli remained between 160 and 170 GPa, and the hardness values were 12 and 13 GPa, respectively. The thin film deposited at 200 °C had a modulus of ≈150 GPa, and the hardness was approximately 10 GPa.

### 3.5. Surface Morphology

**Figure 8:**
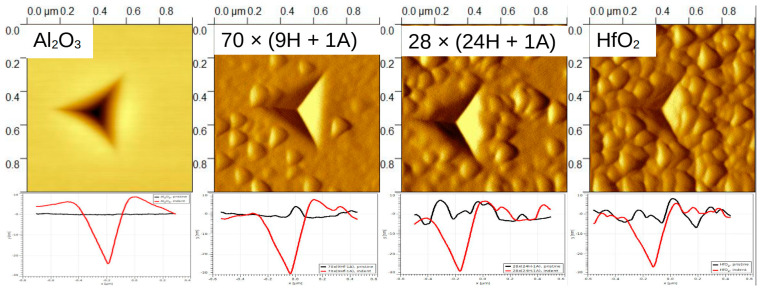
SPM images and respective horizontal profiles of films deposited at 300 °C. The black line indicates plane surface, while red is horizontal profile across the indent. The residual depth of the indent on Al_2_O_3_ was approximately 30 nm, and was similar to depths for other objects.

SPM images (created by using the Gwyddion 2.62 freeware program) sized 1 × 1 µm and 5 × 5 µm were taken of some samples to confirm or deny any pile-up or surface roughness effects. In Figure 8, it can be seen that the amount of surface features increased with the relative increase in HfO_2_ content. (A closer look at 28 × (24H + 1A) can be seen in Supplementary part S4, Figure S4) The profiles, presented at the bottom, show that pure, pristine Al_2_O_3_ had a smooth surface (black line), and the surface roughness increased with the increase in HfO_2_ content. Profiles were taken across a sample. The residual depth of the indent on Al_2_O_3_/Si was approximately 30 nm (respective indentation force 1.5 mN) and was similar to depths for other objects. In the case of alumina thin film, a rise on the indent (red line) edges can be seen, which is an indication of material pile-up around the indent mark. The height of the piled-up material on the left side of the indent was approximately 9 nm. This is a considerable amount compared to the total indentation depth of 70 nm. Therefore, in the case of alumina, only the first few indentation datapoints could be representative of the material system. (Note that the dimensions of the *x* and *y* axes in the profile images differ; *y* is in tens of nanometers, *x* in hundreds.)

**Figure 9:**
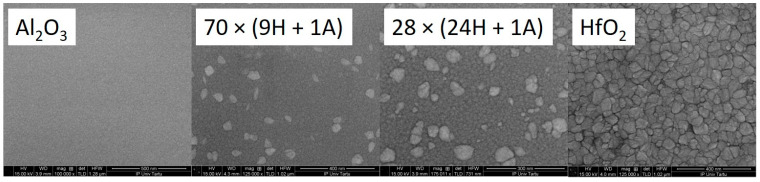
SEM images of selected thin films deposited at 300 °C. The presented area is approx. 1 × 1 micrometer for all samples.

As the tip of the triboindenter is rather blunt (radius approximately 100–150 nm), the SPM images provide a surface morphology description that is rather like a pseudo-surface. Scanning electron microscope images of the same samples were taken to supplement SPM (Figure 9). It can be seen that the surface gets rougher as the amount of deposited HfO_2_ increases. This confirms the previously deduced trend. Micrographs indicate that, in the case of hafnia, for instance, there are grains of crystallites rather than three-sided pyramids on the surface. It is reasonable to consider the triangular features on SPM images as having a convoluted indenter tip shape.

The doped thin films have different morphology compared to pure hafnia surface. Note that the alumina-containing samples have grains alternated with smoother regions.

## 4. Discussion

**Figure 10:**
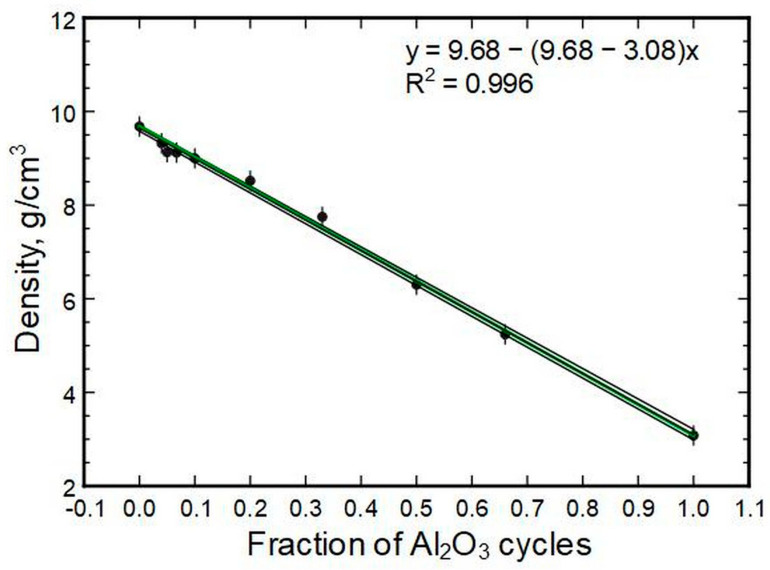
Thin film XRR densities in relation to the Al_2_O_3_ deposition cycle fractions.

The densities of the thin films correlate linearly to the compositions (Figure 10). The best fit had a coefficient of determination over 0.99. This is visualized by the narrow band of deviations in fit parameter errors, also plotted in the image. A model that has hafnia and alumina densities of 9.68 g/cm^3^ and 3.08 g/cm^3^, respectively, as inputs is also shown. The model itself is a derivation of a simple rule of mixture known from composite material theory. The Voigt rule of mixture could be used to predict properties of material systems (mixtures), including density.

During nanoindentation, some influence from the substrate could be expected at 10 nm of tip displacement. At the same time, the properties of the substrate are similar to those of the thin films. Therefore, it could be proposed that the substrate influence was reduced compared to, for instance, that of a substrate possessing a hardness of 20 GPa or a modulus of 340 GPa. This suggests that the values given at approximately 10 nm displacement actually closely resemble those of a thin film.

In several cases, a sudden reduction in hardness can be seen. For pure alumina, this happened at approximately 50 nm of displacement. It has been shown in previous publications that this could be a consequence of coating delamination or cracking [45,46,47,48]. Considering that the pop-in happened during indentation and the SPM images show some material pile-up near the indents, it is likely that the pile-up was caused by thin film delamination and buckling.

Nanocrystals could reinforce a material and increase its hardness, modulus, and fracture toughness. These effects have long been known, for instance, in glass-ceramics research [49,50,51,52,53,54,55]. The amount of crystalline phase and actual phase composition also influence mechanical properties [49,50,51,52]. It has been shown that the appearance of softer phases can reduce hardness but might not ultimately affect the modulus [49]. Also, the modulus can reduce with an increase in crystallinity without having a significant influence on hardness [51].

**Figure 11:**
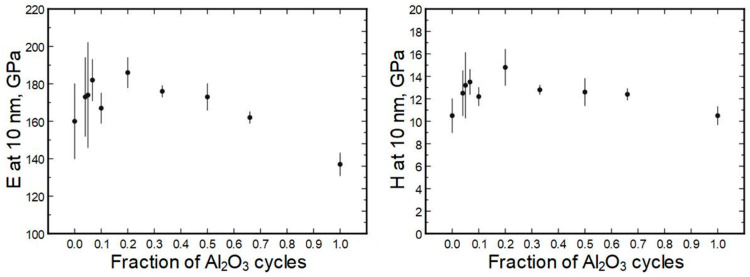
Thin-film moduli in relation to the alumina cycle fractions.

Overall, the deposition recipe or respective elemental composition did not uniformly correlate with hardness or modulus (Figure 11), especially if measurement errors were considered. The general trend seems to be that the doped films had a higher modulus/hardness than pure single oxides.

On average, 140 × (4H + 1A) has a hardness of approximately 15 GPa and a modulus of 184 GPa, making it the hardest and stiffest thin film. Currently, the cause is not clear. Based on effects seen in glass-ceramics, it could be speculated that 140 × (4H + 1A) has a higher hardness and modulus due to the appearance of some short-ordered crystallinity. In the case of 47 × (14H + 1A), it could be that there was some increase in the crystalline phase volume part, compared, for instance, with 70 × (9H + 1A), which was softer. The next mixtures already show different phase compositions based on diffraction patterns. The appearance of different phases, such as monoclinic and cubic/tetragonal in sample 35 × (19H + 1A), could create additional defective interphases around crystallite grains, which could have a detrimental effect on the mechanical properties. This would explain the reduction in hardness/modulus in samples 35 × (19H + 1A) and 28 × (24H + 1A) compared to 47 × (14H + 1A).

**Figure 12:**
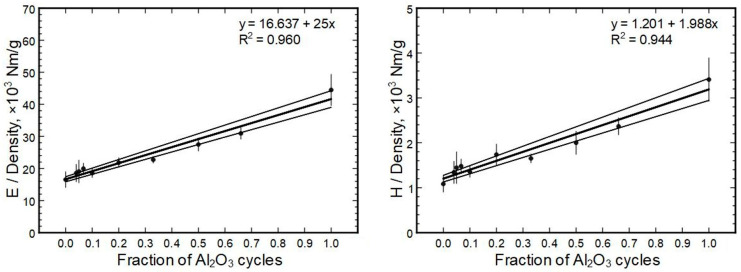
Specific modulus and hardness in relation to the deposited Al_2_O_3_ cycle fractions.

A better correlation could be drawn between the specific modulus (modulus divided by density) or specific hardness and the alumina cycle fraction (Figure 12). Here again, a linear correlation was suitable to model the data. In principle, the best-fit line in both cases goes through all data points, but the scatter is wider. Therefore, the equations shown in Figure 12 have approx. 5% error.

### 4.1. Surface Morphology Influence on Nanoindentation

**Figure 13:**
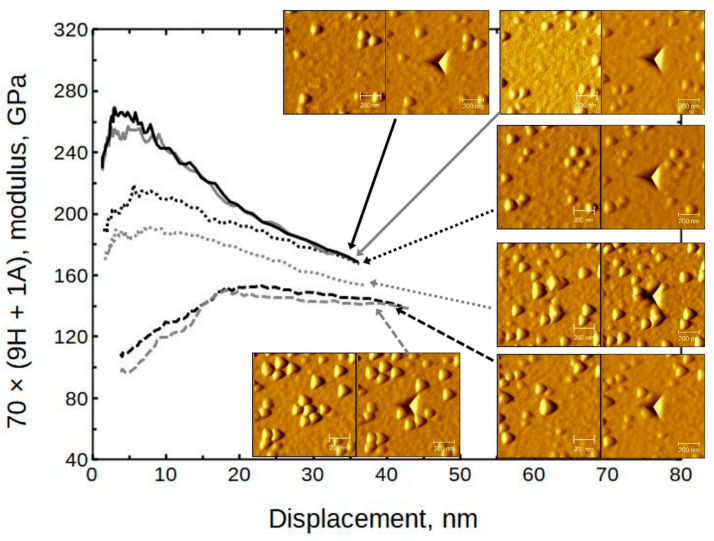
The influence of surface morphology to modulus; sample 70 × (9H + 1A). The paired SPM images show surface before and after indentation.

Several areas with different morphologies were selected by using the indenter SPM capability. Indentations were performed on grainy and other areas of sample 70 × (9H + 1A), grown at 300 °C (Figure 13). The paired images show the surface before and after indentation, respectively. It can be seen that in the case in which the indent is made on a plane surface, the moduli are rather high (near 250 GPa). If the indent is basically on top of the grains, the moduli drop to the 120 GPa level. If the indent is close to some grains but not directly on top, the results tend to be intermediate. The defect density is probably higher near grains. Therefore, a reduction in mechanical resilience should be expected. Any enhancing effects that could be attributed to, for instance, a dislocation movement impediment, were not noticed in any of the samples.

**Figure 14:**
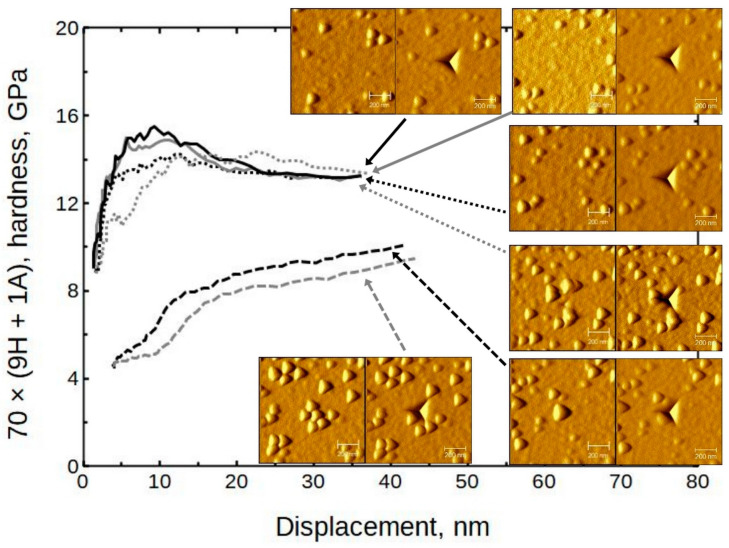
The influence of surface morphology to hardness; sample 70 × (9H + 1A). The paired SPM images show surface before and after indentation.

The corresponding hardness values have a similar tendency (Figure 14), but not as consistent as in the case of the moduli. Note that the presented results are from the same locations.

### 4.2. Correlation of Refractive Index to Composition

The refractive indexes *n* and compositions correlate well within the initial batch of thin films deposited at 300 °C. That batch was used to create a relationship between the two-term Sellmeier formula (for refractive index) and the compositions. In WinElli II v2.2.0.7 software, the Sellmeier parameters *A* and *B* are given as:

$$n\left(\lambda \right)=\sqrt{1+\frac{\left(A^{2}-1\right)\lambda^{2}}{\lambda^{2}-B}}$$

where λ is wavelength in micrometers; the formula considers the imaginary part to be zero.

**Figure 15:**
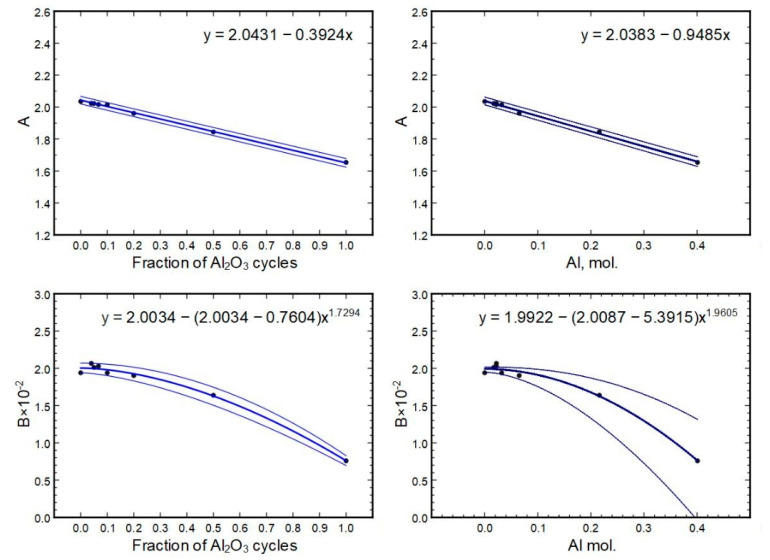
Sellmeier parameters in relation to the thin film compositions.

The relations of *A* and *B* to their respective compositions can be seen in Figure 15. The best-fit line is given as the respective formula on a graph. The error range of a fit was calculated from the formula parameter errors and is depicted as minimum and maximum lines (thinner lines on the graphs).

Based on the equations shown in Figure 15, parameters for samples 233 × (1H + 2A) and 233 × (2H + 1A) were calculated (using the cycle part formulas), and respective indexes *n* versus wavelength were constructed and compared to direct measurements to check the predictive power of the equations. The results are summarized in Table 3.

**Figure 16:**
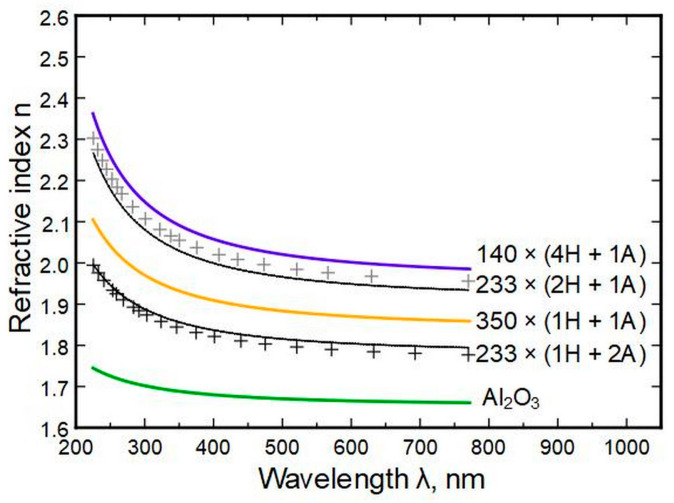
Measured data (crosses) and modeled results (black and grey lines) based on derived Sellmeier parameters.

The initial derivation of A and B values from measurements had an accuracy of approximately 1%, which has to be added/subtracted from the Table 3 minimum/maximum values. Figure 16 shows the results of the measured data (black and grey crosses) and the predictions from models (black and grey lines) as minimum and maximum values. It can be seen that the measurements fit the model within error.

## 5. Conclusions

Aluminum and hafnium oxides were atomic-layer-deposited using metal–organic precursors with oxygen plasma. Plasma ALD of aluminum oxide resulted in stochiometric oxide. ALD of hafnium oxide had excess oxygen in the thin film.

The linear correlation between density and composition means that a simple rule of mixture, known from composite material theory, could also be used to model the results. The agreement between experimental and theoretical approaches was very high. This is an indication that a simple rule of mixture can be used at the nanoscale for density predictions/calculations. In principle, the same applies to specific modulus and hardness, but with a somewhat higher error.

The alumina addition changes crystallography and surface morphology even at 2 mol.% levels. If metastable phases (cubic, tetragonal) are of interest, the hafnium to aluminum cycle ratio should be from approximately 8 ÷ 1 to 15 ÷ 1. At lower ratios, the films tend to be X-ray amorphous, and at higher ratios, hafnia starts to form a monoclinic phase.

The doped thin films had ≈30% higher hardness and modulus values compared to those for alumina. The highest hardness and modulus values were measured for thin films over with 6 mol.% of Al in the thin film. Higher surface roughness hindered analysis and generated larger deviations in the modulus and hardness results. The areas in-between grains had approximately 30% higher modulus (≈240 GPa) and approximately 25% higher hardness (≈15 GPa) compared to the averaged results (≈170 GPa and ≈12 GPa, respectively) of the same sample. The change in material phase composition, increase in interfaces between them, have detrimental effects on the mechanical properties.

Aluminum oxide has a refractive index of approximately 1.70 ± 0.04 in the spectral range of 770–225 nm. This steady value could be employed in the practical design of optical coatings. HfO_2_ had a refractive index in the same wavelength range of 2.07 to 2.33, respectively. A change in the hafnia to alumina deposition cycle ratio changed the refractive index but had a minute effect if the alumina amount was below 4 at.%. The indexes can be predictively computed from composition data by using Sellmeier’s formula, which helps select a suitable coating for an optical system. The models have errors of up to ±3%.

Overall, HfO_2_ with Al_2_O_3_ dopant provides the possibility to develop relatively hard and stiff thin films with tunable optical properties, which could be used in micro-electromechanical systems, for instance.

## Figures and Tables

**Table 1:** The elemental contents of Al_2_O_3_-doped HfO_2_ thin films (deposited at 300 °C if not noted otherwise) measured by XRF.

Deposition Scheme	Amount of an Element, µg/cm^2^			Al at.%
	Hf	Al	O	
700 × A	0	8.66 ± 0.02	7.7 ± 0.2	40.1
233 × (1H + 2A)	18.3 ± 0.2	7.00 ± 0.02	10.0 ± 0.2	25.9
350 × (1H + 1A)	29.7 ± 0.2	6.86 ± 0.02	12.1 ± 0.2	21.6
233 × (2H + 1A)	46.9 ± 0.2	5.09 ± 0.02	14.7 ± 0.3	13.8
140 × (4H + 1A)	55.8 ± 0.4	2.22 ± 0.03	13.9 ± 0.5	6.5
70 × (9H + 1A), 200 °C	60.2 ± 0.4	1.34 ± 0.02	14.8 ± 0.5	3.8
70 × (9H + 1A)	65.3 ± 0.3	1.16 ± 0.02	14.8 ± 0.5	3.2
70 × (9H + 1A), 400 °C	53.8 ± 0.4	1.24 ± 0.02	12.7 ± 0.5	4.1
47 × (14H + 1A)	64.8 ± 0.3	0.74 ± 0.02	13.8 ± 0.5	2.2
35 × (19H + 1A)	66.6 ± 0.4	0.59 ± 0.02	14.3 ± 0.6	1.7
28 × (24H + 1A)	64.6 ± 0.3	0.70 ± 0.02	13.8 ± 0.5	2.1
700 × H	78.15 ± 0.3	0	16.21 ± 0.6	

**Table 2:** Al_2_O_3_-doped HfO_2_ thin-film thickness and density, derived from XRR measurements.

Formula	Thickness, nm	Density, g/cm^3^
700 × A	63.8	3.08
233 × (1H + 2A)	60.8	5.24
350 × (1H + 1A)	62.4	6.30
233 × (2H + 1A)	69.1	7.75
140 × (4H + 1A)	70.2	8.52
70 × (9H + 1A), 200 °C	75.5	8.66
70 × (9H + 1A)	73.5	9.10
70 × (9H + 1A), 400 °C	62.2	9.14
47 × (14H + 1A)	70.9	9.12
35 × (19H + 1A)	69.2	9.13
28 × (24H + 1A)	67.3	9.32
700 × H	78.8	9.68

**Table 3:** Sellmeier parameters and respective limits from the A and B correlations to the compositions.

Deposition Scheme	Best Fit A	Min/Max A	Best Fit B (×10^−2^)	Min/Max B (×10^−2^)
233 × (1H + 2A)	1.782	1.774/1.789	1.387	1.293/1.474
233 × (2H + 1A)	1.912	1.907/1.918	1.830	1.739/1.884

## Data Availability

The data that support the findings of this study are available from the corresponding author upon reasonable request.

## Supplementary Materials

The following supporting information can be downloaded at: https://www.mdpi.com/article/10.3390/nano13101607/s1, Figure S1: X-ray reflectance (XRR) of all samples; Figure S2: Refractive indexes of all samples; Figure S3: Nanoindentation modulus and hardness results of all samples; Figure S4: Close-up view of sample 28 × (24H + 1A) SPM image and respective cross-profiles.
