# Supplementary material for: Hardness, Modulus, and Refractive Index of Plasma-Assisted Atomic-Layer-Deposited Hafnium Oxide Thin Films Doped with Aluminum Oxide
Source: Nanomaterials (Basel). 2023 May 10;13(10):1607. doi: 10.3390/nano13101607 (PMC10223527; doi:10.3390/nano13101607)
Supplement: Supplementary file 1 [file nanomaterials-13-01607-s001.zip › nanomaterials-2373864-supplementary.pdf]

Supplementary S1

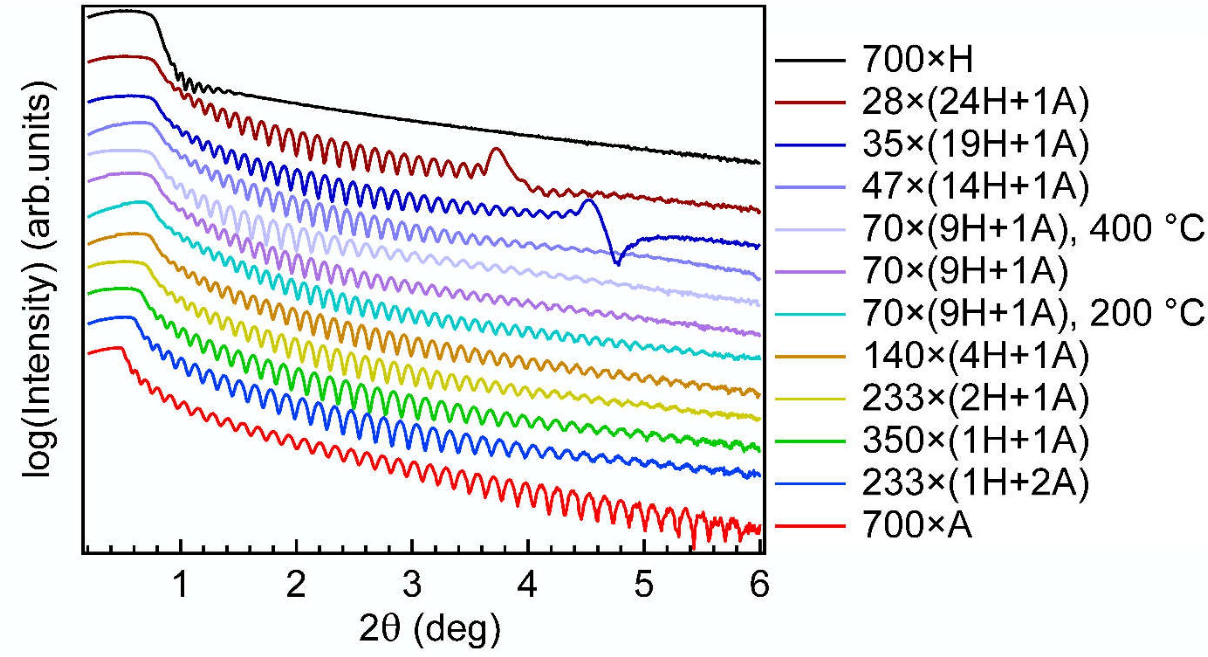

Figure S1. X-ray reflectance of all samples.

Supplementary S2

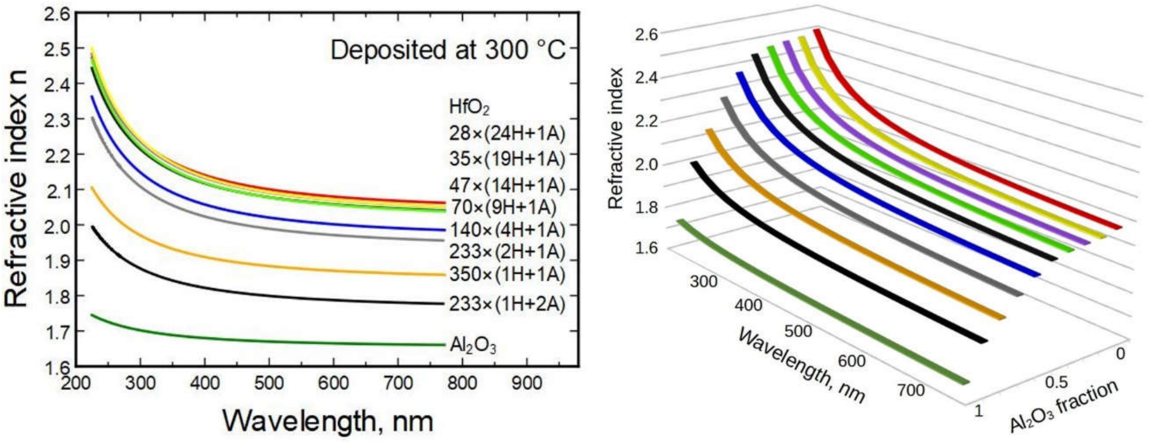

Figure S2. Refractive indexes of all samples.

Supplementary S3

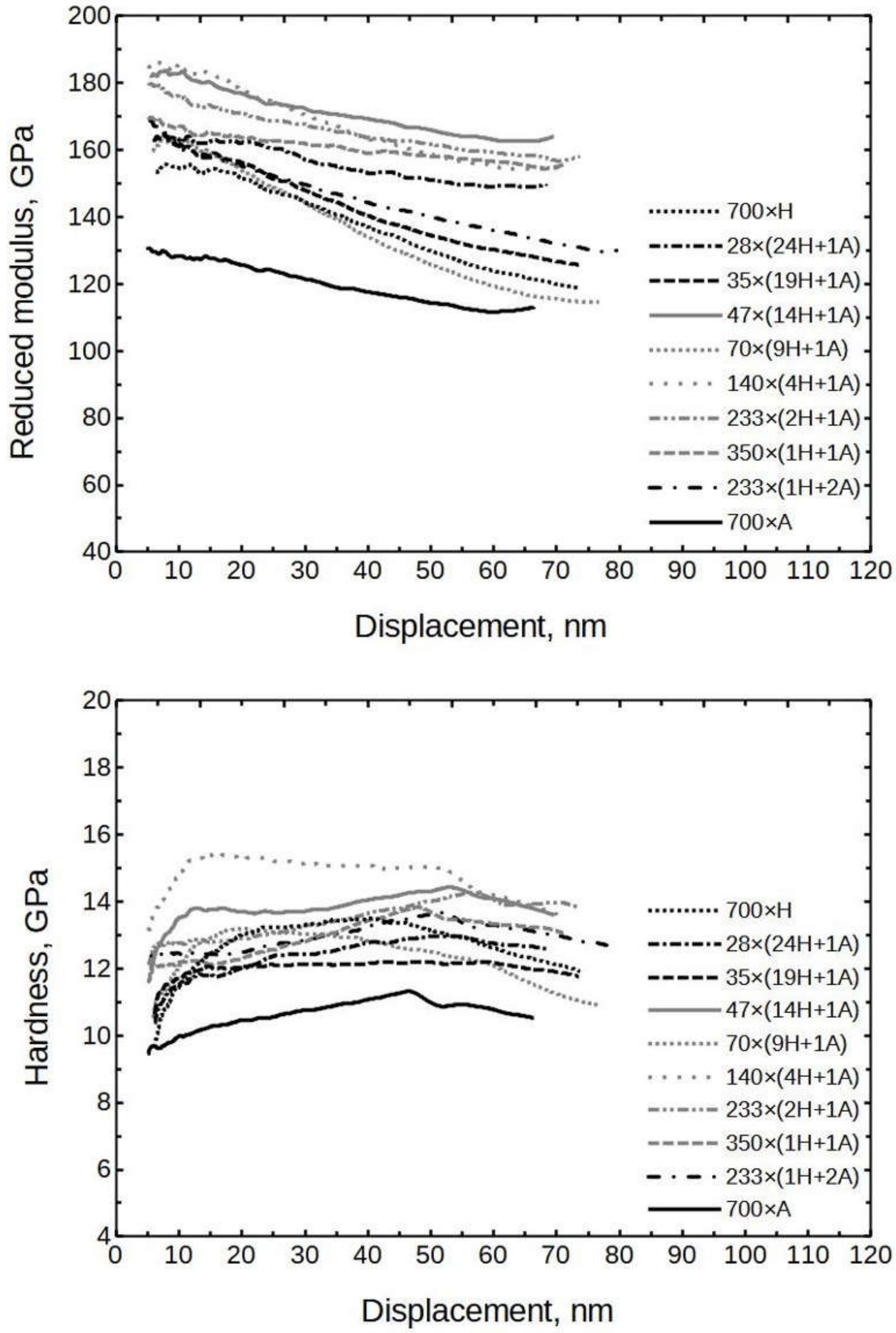

Figure S3. Nanoindentaion modulus and hardness results of all samples.

Supplementary S4

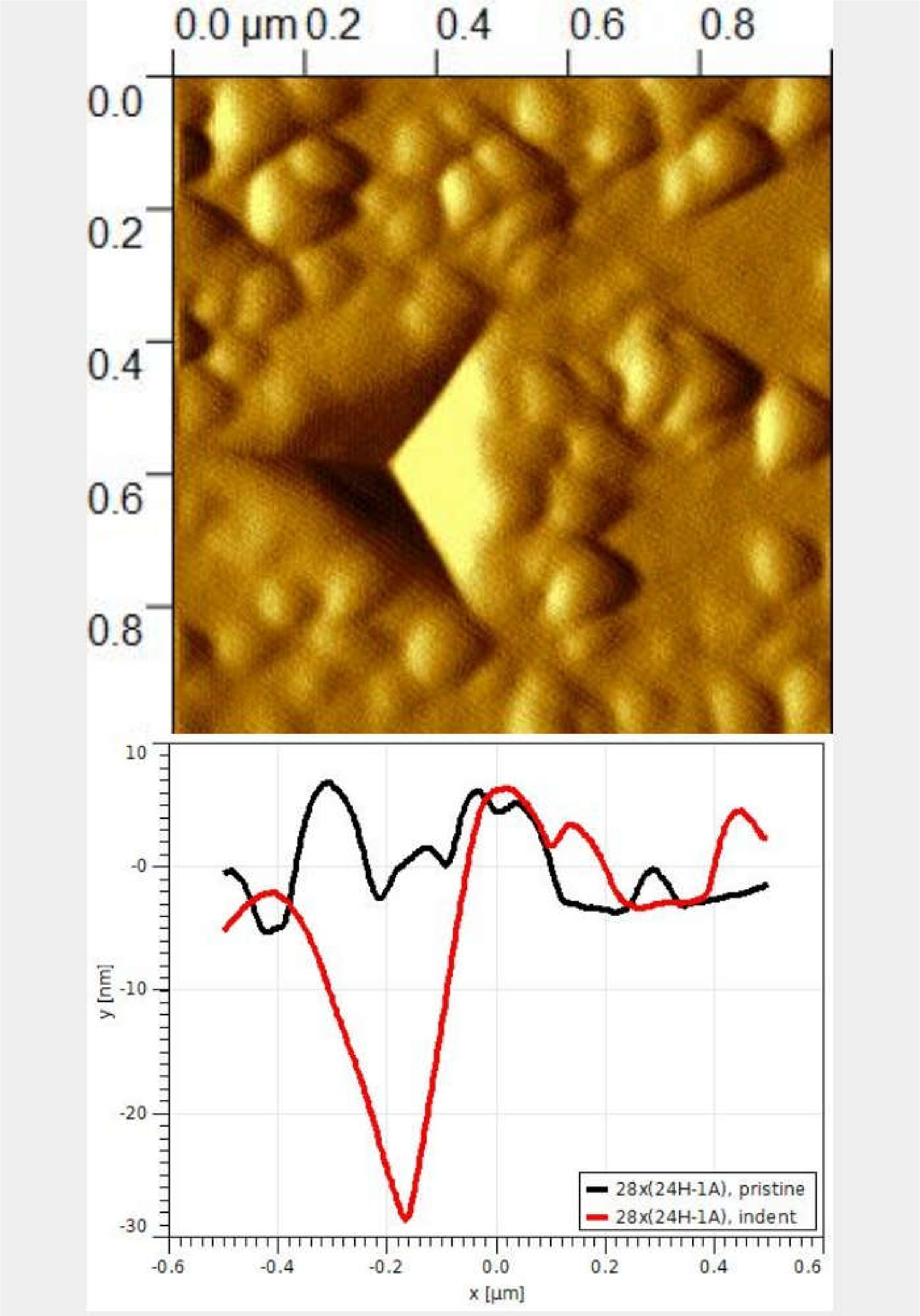

Figure S4. Close-up view of sample 28(24H-1A) SPM image and respective cross-profiles.
